# Supplementary material for: Nano-Particles Carried by Multiple Dynein Motors Self-Regulate Their Number of Actively Participating Motors
Source: Int J Mol Sci. 2021 Aug 18;22(16):8893. doi: 10.3390/ijms22168893 (PMC8396316; doi:10.3390/ijms22168893)
Supplement: Supplementary file 1 [file ijms-22-08893-s001.zip › ijms-1287093-sup-final.pdf]

Supplementary Material

1. NP Synthesis and Characterization

The NP synthesis process entails several consecutive steps, where at each step, a single component is added (see Figure 1A, main text). Bare NPs saturated with Neutravidin were conjugated with Biotin-PEG-thiol spacers, then incubated for a short time in a solution containing SV40 T large antigen NLS peptides at variable concentrations; see Materials and Methods section. The resulting NPs were characterized using Cryo-TEM, dynamic light scattering (DLS), and zeta potential. In addition, adsorption isotherms from UV-Vis experiments yield the surface density and the mean number of grafted Biotin-PEG-thiol that are end-conjugated by NLS (PEG-NLS), $\langle N \rangle$. The latter is transformed into a mean anchoring distance between neighboring PEG-NLSs, ξ^* (Figure 1B, main text). For the experiments we use bare NPs of different diameters. We use the same total surface area of bare NPs in the experiments. This guarantees that the mean anchoring distance is independent of the NPs diameter (data not shown). Next, the NPs were incubated in (Hela) cell extract, allowing the recruitments of α- and β-importins, dynactin, and *mammalian* dynein; the recruitment of the importins, dynactin, and dynein is verified *via* Western Blot (Figure 1C, main text). The particles are washed from the excess cell extract, incubated in an ATP solution, and then injected into a flow cell in which MTs are adsorbed and immobilized on a glass surface.

Several measurements were carried out to verify the NPs integrity and to study the various components effect on the NP characteristics, i.e., the complete integration of the NP and the different components. First, to study the effect of the grafted Biotin-PEG-thiol, we examined the distances between adjacent NPs using Cryo-TEM [56]. We compared cryo-TEM microscopy images of a control group without grafted Biotin-PEG-thiol (Figure S1A) and NPs with grafted Biotin-PEG-thiol molecules (Figure S1B). The images show an NPs in which inter-particle distances depend on the absence or presence of grafted Biotin-PEG-thiol molecules. Without Biotin-PEG-thiol, the mean spacing between the NPs is 10.5 ± 7 nm (mean $\pm$ STD), typically twice the diameter of a Neutravidin molecule [76], and with a $M_{\mathrm{w}} = 5$ kDa Biotin-PEG-thiol, it is 26 ± 5 nm (mean $\pm$ STD), which is much larger than the theoretical value of the (free polymer) gyration radius: $R_{\mathrm{g}} = 2.27$ nm (see SI.2 below). This is consistent with weak attractive entropic forces (e.g., depletion attraction) working against the entropic repulsion resulting from the anchored chains [68]. Note that the black dots on the particle's surface are the Neutravidin molecules (Figure S1). Since Neutravidin diameter is about 5 nm [76], and since the Neutravidins are closely packed on the NP surface, we conclude that the anchored Biotin-PEG-thiol molecules are effectively in the so-called "mushroom regime" [77].

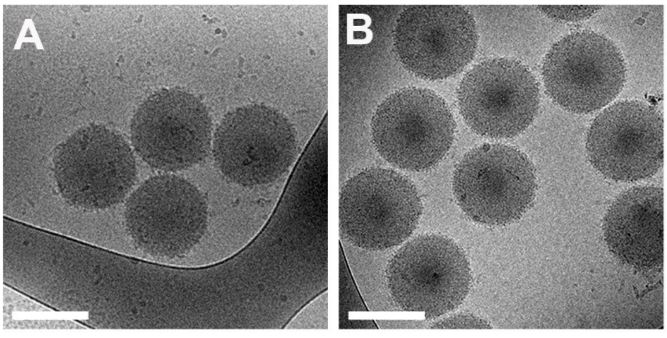

Figure S1. Cryo-TEM microscopy images of Neutravidin-coated NPs without (A) and with (B) grafted $M_{\mathrm{w}} = 5$ kDa Biotin-PEG-thiol. Bare NPs mean diameter is 196 nm. Bars are 200 nm.

To follow the NP decoration during the various stages, we performed dynamic light scattering (DLS) experiments. The data show a significant increase in the NP hydrodynamic diameter (D_{h}) during the first two decoration steps: 245 ± 10 nm (Stage I) and

410 ± 4 nm (Stage II) compared to the bare NPs, which have D_h = 198 ± 3 nm (all values correspond to mean ± STD). At the third step (NLS binding), a slight decrease is detected, confirming the Biotin-PEG-thiol binding to the NP.

Next, to conclude on the NP charge at the various decoration steps, we examined the zeta potential [57] (see Materials and Methods section). The zeta potential is highly negative for the bare NPs and has a value of −41.4 ± 1 mV (mean ± STD) (i.e., the bare NPs are negatively charged), and it remains negative, but gradually decays (in absolute values) as NP decoration advances. Regarding stages I and III, this trend is consistent with the assumption that both the Neutravidin and NLS are positively charged; thus, a reduction of the (absolute value of) the zeta potential is expected after their binding. At the end of stage III (NLS binding), the zeta potential equals −9.6 ± 0.5 mV (mean ± STD).

Finally, to determine the mean number of bound PEG-NLS molecules per NP, $\langle N \rangle$, and the mean anchoring distance between adjacent PEG-NLS molecules, ξ^*, we carried out UV-Vis absorption experiments using fluorescently labeled NLS, TAMRA-NLS (see Materials and Methods section, main text). We incubated Biotin-PEG-thiol-grafted NPs with increasing amounts of TAMRA-NLS and measured the absorption of the remaining TAMRA-NLS molecules that *did not* adsorb to the NPs (i.e., supernatant). From the absorbance, we deduced the number of TAMRA-NLS molecules that *did* adsorb to the NPs, from which we can deduce the mean number of bound PEG-NLS molecules per NP:

$$\sum N = 6.02 \cdot 10^{23} \times V \times \left([\mathrm{NLS}] - [\mathrm{NLS}]_{\mathrm{supernatant}}\right) \quad \text{(S1)}$$

$$\langle N \rangle = \frac{\sum N}{N_{\mathrm{NP}}} \quad \text{(S2)}$$

Where V is solution volume, [NLS] is the concentration of TAMRA-NLS in the incubation solution, $[\mathrm{NLS}]_{\mathrm{supernatant}}$ is the concentration of TAMRA-NLS that remained in the bulk and did not adsorb to the NPs, $6.02 \cdot 10^{23}$ is the Avogadro number, $\sum N$ is the total number of TAMRA-NLS molecules that were adsorbed to the NPs surface, N_{NP} is the total number of NPs – shown in Figure S2 the results for bare NPs of 20nm in radius. Similar results are obtained with bare NPs of 196 nm in diameter (data not shown).

The dependence of $\langle N \rangle$ on the concentration of TAMRA-NLS (NLS in Figure S2) follows a Langmuir-like isotherm. We used the parameters extracted from the fit to estimate the mean number of bound motors per NP for [NLS] of 0.05 and 0.025 μM, which are below the detection limit of our UV-Vis set-up (grey dot in Figure S2). Our motility assays are performed with [NLS] varying between 0.025 and 0.3 μM, implying that $\langle N \rangle$ varies between 5 and 37.

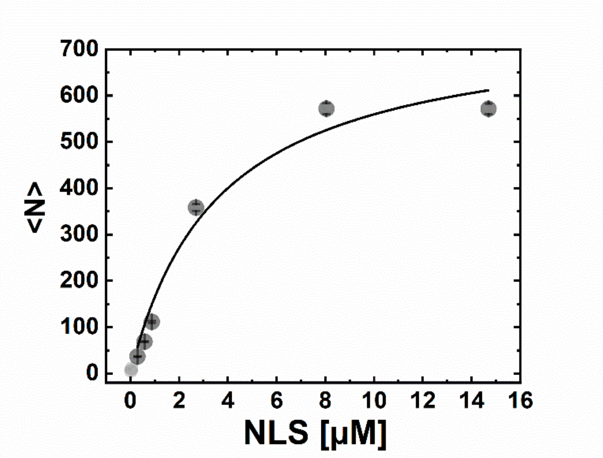

Figure S2. Dependence of the mean number of grafted PEG-NLS, $\langle N \rangle$, against the concentration of TAMRA-NLS (marked as NLS) follows a Langmuir-like isotherm (grey dots – experimental data, line - fit to the experimental data; R^2 = 0.96). The two bright grey dots correspond to extrapolated

values of $\langle N \rangle$ calculated from the fit, for [NLS] = 0.025 and 0.05 μM. Error bars indicate the standard deviations for 3 experiments. Bare NPs mean radius is 20 nm.

Finally, we deduce the mean anchoring distance between adjacent PEG-NLS molecules, ξ^*, from $\langle N \rangle$ assuming each PEG-NLS molecule occupies a square lattice ξ^{*2},

$$\xi^* = \sqrt{\frac{4\pi R^2}{\langle N \rangle}} \tag{S3}$$

where $R = 25$ nm is the mean radius of the Neutravidin-coated NPs. The dependence of ξ^* on [NLS] is depicted in Figure 1B in the main text.

2. Estimation of the PEG contour length, C_L, and Radius of Gyration, R_g

In our motility experiments, we use biotin polyethylene glycol thiol (Biotin-PEG-thiol) with a molecular weight of 5 kDa. The molar mass of each ethylene glycol subunit, the building block of the Biotin-PEG-thiol polymer spacer (marked by the red rectangle, Figure S3), is $44.05 \frac{\mathrm{g}}{\mathrm{mol}}$. The number of repeating ethylene glycol subunits, n, for a 5 kDa Biotin-PEG-thiol, is $n = \frac{5000}{44} = 114$.

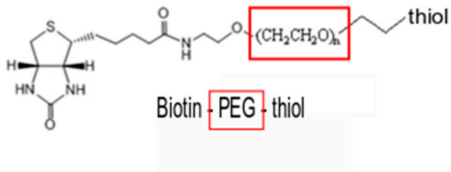

Figure S3. Molecular structure of the biotin polyethylene glycol thiol (Biotin-PEG-thiol) used. n refers to the number of ethylene glycol units per PEG polymer.

To estimate the Biotin-PEG-thiol contour length C_L and radius of gyration, R_g, we use the length of an ethylene glycol subunit d_EG [69] and the number of ethylene glycol subunits, n, as follows [70-72]:

$$C_\mathrm{L} = d_\mathrm{EG} \times n \tag{S4}$$

$$C_\mathrm{L} = N_\mathrm{a} \times a = d_\mathrm{EG} \times n \tag{S5}$$

$$N_\mathrm{a} = \frac{d_\mathrm{EG} \times n}{a} \tag{S6}$$

$$R_0 = \sqrt{N_\mathrm{a}}a \tag{S7}$$

$$R_\mathrm{g} \cong \frac{R_0}{\sqrt{6}} = \frac{\sqrt{N_\mathrm{a}}a}{\sqrt{6}} = \frac{\sqrt{d_\mathrm{EG} \times n \times a}}{\sqrt{6}} \tag{S8}$$

Where R_0 is the polymer end-to-end distance (assuming that the PEG polymer behaves like a Gaussian chain[73,74], $a = 0.76$ nm is the PEG Kuhn length [73,75], N_a is the number of Kuhn length segments, and $d_\mathrm{EG} = 0.358$ nm [69,73]. Using these parameters we find that $C_\mathrm{L} = 40.8$ nm and $R_\mathrm{g} = 2.27$ nm.

3. Summary of Experimental Results.

Table S1. NPs experimental mean velocities, run-times, and run-lengths (mean ± SEM). υ is the absolute velocity, υ_x is the longitudinal velocity, υ_y is the transverse velocity, τ_p is the processivity time, λ^* is the absolute accumulated distance that the NP covered regardless of the motion direction, and λ is the total longitudinal run length in the direction of the MT minus-end. Bare NPs mean radius is 20 nm.

	**I**	**II**	**III**	**IV**	**V**
[NLS] [μM]	0.025	0.05	0.05	0.3	0.3
$\langle N \rangle$	5.2	10.4	10.4	36.6	36.6
[CE] [mg/mL]	3.4	3.4	3.4	3.4	6.8
Number of steps	80	62	428	134	536
$\langle \lvert\upsilon\rvert \rangle$ [nm/s]	905±18	793 ± 16	847 ± 33	493 ± 23	320 ± 20
$\langle \upsilon_x \rangle$ [nm/s]	815±68	769 ± 49	619 ± 39	422 ± 26	72 ± 22
$\langle \upsilon_x \rangle \mid \upsilon_x > 0$ [nm/s]	939±57	783 ± 48	687 ± 18	476 ± 22	253 ± 20
$\langle \upsilon_x \rangle \mid \upsilon_x < 0$ [nm/s]	–305±141	-91 ± 0	-609 ± 160	-224 ± 76	-319 ± 43
$\langle \upsilon_y \rangle$ [nm/s]	1.7 ± 25	-0.07 ± 24	206 ± 27	67 ± 16	39 ± 10
$\langle \upsilon_y \rangle \mid \upsilon_y > 0$ [nm/s]	190±24	173 ± 23	278 ± 24	92 ± 13	132 ± 9
$\langle \upsilon_y \rangle \mid \upsilon_y < 0$ [nm/s]	–152±23	-143 ± 16	-299 ± 62	-277 ± 104	-162 ± 18
$\langle \tau_p \rangle$ [s]	2.1±0.4	3.0 ± 0.8	3.9 ± 0.5	11.7 ± 1.4	23.3 ± 4.1
$\langle \lambda^* \rangle$ [nm]	2200±300	2600 ± 540	3800 ± 720	4900 ± 750	7700 ± 1100
$\langle \lambda \rangle$ [nm]	1700±260	2300 ± 480	2800 ± 610	4300 ± 680	1700 ± 600
Fractions of forward & backward steps	0.9 f ∣ 0.1 b	0.98 f ∣ 0.02 b	0.9 f ∣ 0.1 b	0.92 f ∣ 0.08 b	0.67 f ∣ 0.33 b
Fraction of Right & Left-handed steps	0.45 r ∣ 0.55 l	0.45 r ∣ 0.55 l	0.92 r ∣ 0.08 l	0.93 r ∣ 0.07 l	0.67 r ∣ 0.33 l

Table S2. Standard deviations, STDs, for the experimental velocity, run-time, and run-length values of systems I to V. υ is the absolute velocity, υ_x is the longitudinal velocity, υ_y is the transverse velocity, τ_p is the processivity time, λ^* is the absolute accumulated distance that the NP covered regardless of its direction, and λ is the total longitudinal run length in the direction of the MT minus-end. Bare NPs mean radius is 20 nm.

	**I**	**II**	**III**	**IV**	**V**
[NLS] [μM]	0.025	0.05	0.05	0.3	0.3
[CE] [mg/mL]	3.4	3.4	3.4	3.4	6.8
#steps	80	62	428	154	573
$\langle \delta \lvert\upsilon\rvert \rangle$ [nm/s]	168	126	682	288	477
$\langle \delta \upsilon_x \rangle$ [nm/s]	605	386	856	326	518
$\langle \delta \upsilon_y \rangle$ [nm/s]	226	192	592	193	234
$\langle \delta \tau_p \rangle$ [s]	1.61	2.13	3.12	6.95	16.24
$\langle \delta \lambda^* \rangle$ [nm]	1055	1436	4496	3610	4340
$\langle \delta \lambda \rangle$ [nm]	932	1277	3809	3750	2390

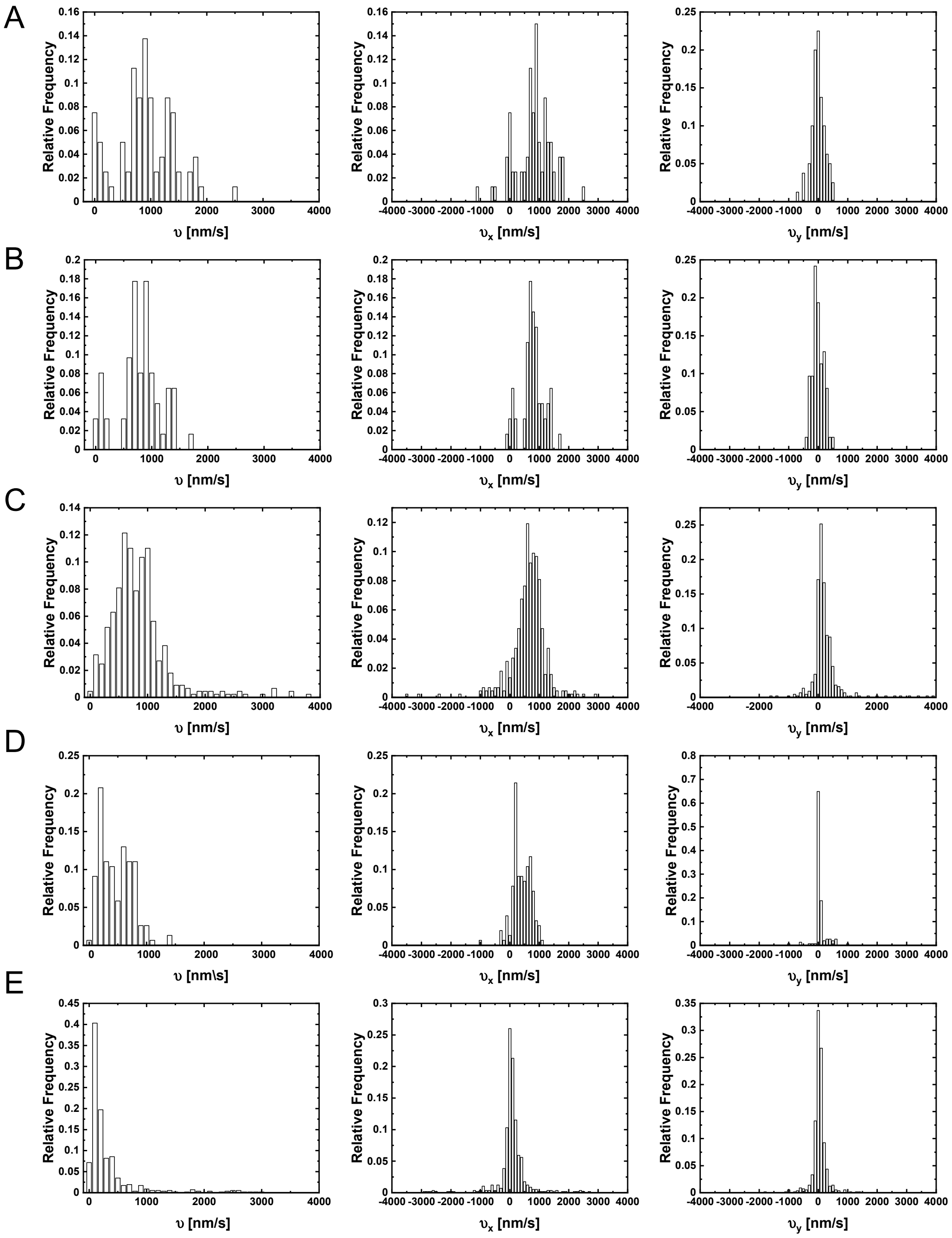

Figure S4. Histogram of the experimental velocities for systems I to V (rows A-E, respectively), where υ is the absolute velocity, υ_x is the longitudinal velocity, and υ_y is the transverse velocity. Bare NPs mean radius is 20 nm.

4. NP motion orientation and estimation of d_{dynein} and $d_{\text{Importins}}$

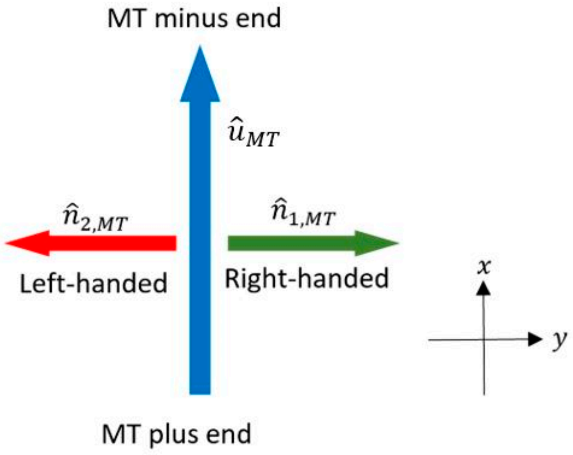

Figure S5. Illustration of the $\hat{\boldsymbol{u}}_{\text{MT}}$ vector (blue arrows) and of the two $\hat{\boldsymbol{n}}_{\text{MT}}$ vectors (green and red arrows). The x-axis is along the MT cylindrical axis of symmetry, and y-axis is orthogonal to it.

As depicted in Figure S5, for each MT or MT unit vectors $\hat{\boldsymbol{u}}_{\text{MT}}$, there are two opposite normal unit vectors that can be assigned, $\hat{\boldsymbol{n}}_{1,\text{MT}}$ and $\hat{\boldsymbol{n}}_{2,\text{MT}}$, which account for positive and negative NP transverse motions, respectively.

d_{dynein} is estimated as follows: The dynein structure, without the tail domain, can be said to consist of three major domains: MTBD, stalk, and AAA+ ring, with the approximated dimensions of 4, 15, and 10 nm, respectively – a total of 30 nm [64,65]. The length of the dynein (d_{dyn}), more or less, equals to the length of the dynein heavy chain. It is known [67] that two-thirds of the chain (C-terminal) spans from the MTBD to the AAA+ ring, and the remaining portion is the "tail" domain backbone (N-terminal). Therefore, a simple calculation leads to a total length, d_{dyn}, of about 45 nm.

$d_{\text{Importins}}$ is taken as 15 nm. However, it is a rough estimation obtained by using Chimera that lies between two conformations: dense and relaxed [66].

5. Experimental mean angular velocity $\langle\boldsymbol{\omega}\rangle$ and helical pitch $\langle\overline{\boldsymbol{H}}\rangle$

Table S3. Experimental data of Figures 5 and S6. Mean angular velocity, $\langle\omega\rangle$, estimated mean helical pitch size, $\langle\overline{H}\rangle$, mean angular velocities for right- ($\langle\omega\rangle|\omega > 0$) and left- ($\langle\omega\rangle|\omega < 0$) handed motions, and mean angular velocity evaluated separately for minus-end directed ($\langle\omega\rangle|v_{\text{x}} > 0$) and plus-end directed ($\langle\omega\rangle|v_{\text{x}} < 0$) longitudinal motions for systems I-IV (mean $\pm$ SEM). Bare NPs mean radius is 20 nm.

System	[NLS] [μM]	[CE] [mg/mL]	$\langle\omega\rangle$ [rad/s]	$\langle\overline{H}\rangle$ [nm]	$\langle\omega\rangle|\omega > 0$ [rad/s]	$\langle\omega\rangle|\omega < 0$ [rad/s]	$\langle\omega\rangle|v_{\text{x}} > 0$ [rad/s]	$\langle\omega\rangle|v_{\text{x}} < 0$ [rad/s]
I	0.025	3.4	-0.02 ± 0.2	$2.6 \times 10^5 \pm 3.1 \times 10^6$	1.27 ± 0.02	-1.01 ± 0.02	-0.11 ± 0.2	0.67 ± 0.8
II	0.05	3.4	0.019 ± 0.2	$2.6 \times 10^5 \pm 2.5 \times 10^6$	1.15 ± 0.02	-0.94 ± 0.02	-0.012 ± 0.2	0.38 ± 0.2
III	0.05	3.4	0.85 ± 0.1	8527 ± 6140	1.76 ± 0.02	-0.93 ± 0.02	1.00 ± 0.1	0.58 ± 0.6
IV	0.3	3.4	0.25 ± 0.03	$1.1\times 10^4 \pm 1980$	0.28 ± 0.01	-0.40 ± 0.1	0.29 ± 0.01	-0.27 ± 0.1

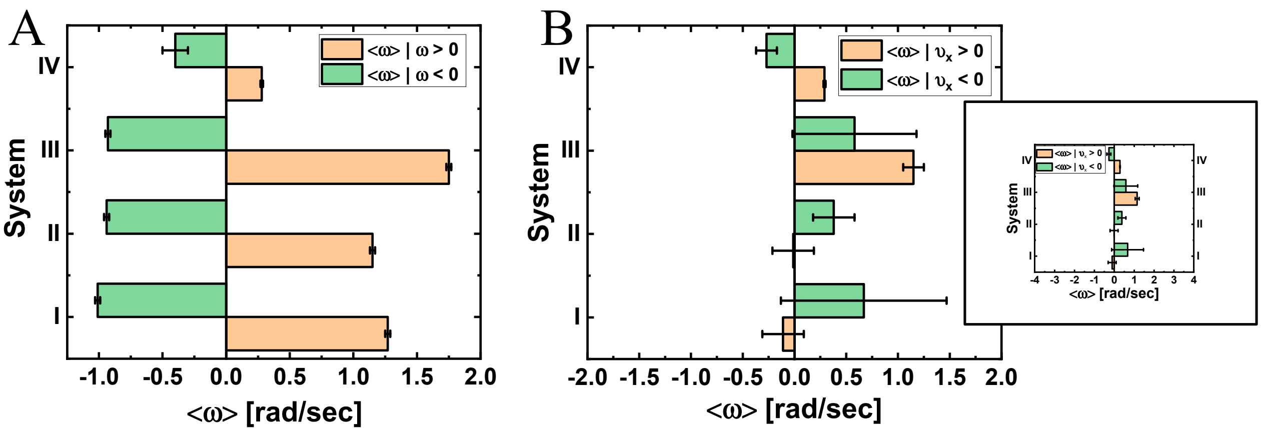

Figure S6. **(A)** Mean right- ($\langle\omega\rangle|\omega > 0$) and left- ($\langle\omega\rangle|\omega < 0$) handed angular velocities. **(B)** Mean angular velocity evaluated separately for minus-end directed ($\langle\omega\rangle|\upsilon_x > 0$) and plus-end directed ($\langle\omega\rangle|\upsilon_x < 0$) longitudinal motions. Inset: same as main figure, but on a larger x-axis scale, like the scale of Figure 9C (theory). The bare NPs mean radius is 20 nm. Raw data for the figure appears in Table S3.

6. Model and Simulation Algorithm

Our simulations are based on the well-known Monte-Carlo (MC) method, see Figure S7. We simulate processes that occur on timescales much longer than the polymer Zimm relaxation time and the NP rotational and translational timescales. All these processes are associated with thermal diffusion, controlling fluctuations, and are also responsible for relaxation towards mechanical equilibrium. These relaxation times are estimated in Supplementary Material Section 6.2.5 for completeness. We simulate the motion of a 20 nm radius NP that is coated with a fixed number of polymers grafted to its surface, each ending with a dynein motor protein. The number of grafted polymers per NP, N_{m}, is related to the particle radius R and the anchoring spacing, ξ, *via* $N_{\mathrm{m}} = 4\pi R^2/\xi^2$. To accurately model the experiment, we distribute the polymers randomly over the NP surface. To reduce computation time, we do not consider the actual dynein structure. Instead, we treat the dynein as a simple cylindrical-like body, with a radius of 10 nm and a height of 60 nm. The height is an approximation that is built from the accumulated dimensions of the dynein components and the $\alpha\beta$ importin complex (Supplementary Material Section 4). The reasoning for the choice of 10 nm radius is explained below.

The MT surface, over which the motors step, is modeled as a cylinder with a 12.5 nm radius. The MT surface is covered with binding sites for the dynein MTBDs. To simulate the motion of the dynein pivot location, we used the MT binding site array for the MTBDs (as presented in previous work [40]) to create an artificial MT-dynein (i.e. dynein pivot) binding site grid. We did it by sampling all the possible configurations of an MTBD pair, and for each of the configurations, we calculated the corresponding location of the dynein pivot.

The simulation algorithm which describes a single NP trajectory, includes a sequence of events, beginning with the binding of a single motor to the MT, and ending when the last motor detaches (henceforth, we use the term "motor" to refer to the dynein-polymer complex). Each simulation iteration (MC-step) involves one of the following competing events that is allowed for each of the motors: (i) motor binding (for the unbound motors), (ii) unbinding (for the bound motors), or (iii) stepping (for the bound motors); the latter might lead to a jamming event as discussed below. The probability of each event i is proportional to its rate, k_{i}. To avoid a non-vanishing, computation time consuming, probability for no occurrence of any of the three events, we choose all event probabilities, for each MC-step, to sum up to unity, such that the probability of an event i is given by $p_{\mathrm{i}} = \frac{k_{\mathrm{i}}}{\sum_{\mathrm{i}} k_{\mathrm{i}}}$. With this choice, the physical time corresponding to an MC-step is $\delta t = \frac{1}{\sum_{\mathrm{i}} k_{\mathrm{i}}}$, implying that this time increment (unlike common MC simulations) varies between different MC steps. For convenience, we separate the events to the above three groups such that the

rate for an event class α is $K_\alpha = \sum_{i\in\alpha} k_i$, with α= (binding, unbinding, and stepping), such that the sum on $i \in \alpha$ runs over the different participating motors; the corresponding probability for each event class α is $P_\alpha = \frac{K_\alpha}{\sum_\alpha K_\alpha}$. Thus, after an event group has been randomly selected with probability P_α, a second random selection is performed for the actual event within the group with probability $p_{i\in\alpha} = \frac{k_{i\in\alpha}}{\sum_{i\in\alpha} k_i}$. For instance, a random selection of a "binding" process (out of the three processes) is followed by the selection of the motor identity out of the unbound motors. The above procedure is entirely equivalent to a random selection of an event without the prior selection of one of the three classes, since $p_i = P_\alpha\, p_{i\in\alpha}$. After each such MC-step, the NP is balanced mechanically by minimizing the free-energy, mimicking the relaxation processes mentioned above that are orders of magnitude faster than the MC timescale (see Supplementary Material Section 6.2.5). Our MC algorithm can be summarized in the following flow chart, Figure S7.

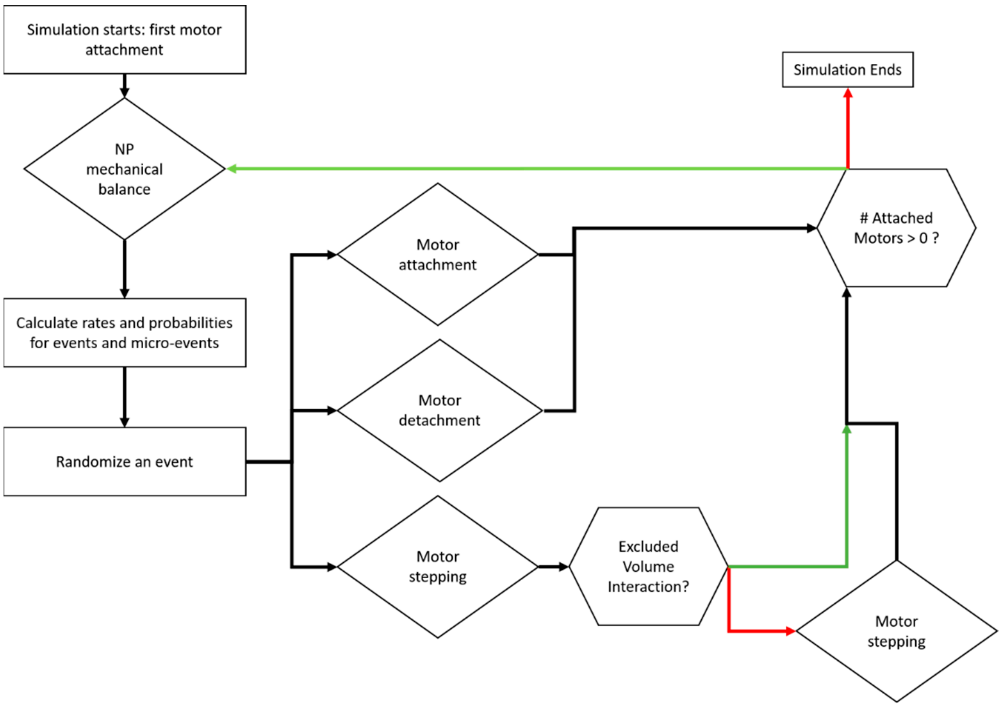

Figure S7. Scheme of the simulation algorithm. Upon a question presented (e.g., "Excluded Volume Interaction?"), green line represents positive answer, and red line represents negative answer.

6.1. Stepping Model for Single Mammalian Cytoplasmic Dynein

In a previous publication [40], we presented a stochastic model for the 2D stepping of single cytoplasmic *yeast* dynein, which shows excellent agreement with experimental results. Here, we use a similar model for single *mammalian* dynein. Although these two motor proteins differ in several aspects, the conversion of our *yeast* dynein stepping model to the *mammalian* case can be achieved using a few assumptions and additional experimental data.

The main assumptions concerning the conversion of the (single) *yeast* dynein stepping model to *mammalian* dynein are: **(i)** Both *yeast* and *mammalian* dynein MTBDs bind to the same binding sites on the MT surface. **(ii)** All the required biological factors, such as bicaudal-D-homolog-2 (BICD2) and dynactin, are at optimal concentrations, allowing to neglect their binding-unbinding kinetics. **(iii)** The stepping vector probability distribution, $P(\vec{\boldsymbol{L}})$, for both dynein types, is written as a product of two functions:

$$P(\vec{\boldsymbol{L}}) = A\, W(\vec{\boldsymbol{L}})\, G(\vec{\boldsymbol{L}}) \tag{S9}$$

where $\vec{\boldsymbol{L}}$ is the dynein step vector (with longitudinal and transverse components L_{x} and L_{y}, respectively), and A is a normalization factor. $W(\vec{\boldsymbol{L}})$ is a motor-specific function that accounts for the stepping model rules [40] and is a symmetric function (i.e., invariant under the transformation $\vec{\boldsymbol{L}} \rightarrow -\vec{\boldsymbol{L}}$); thus, its projection along L_{y}, i.e., $w(L_{\mathrm{y}}) = \int_{-\infty}^{\infty} dL_{\mathrm{x}} W(\vec{\boldsymbol{L}})$, is also a symmetric function of its variable. $G(\vec{\boldsymbol{L}})$ is a force exertion function that includes the internal (longitudinal) motor force, equal in magnitude to the so-called stalling force F_{s}, thus breaking the symmetry between forward (minus-end directed) and backward (plus-end directed) steps. It also may include any external potential $U(\vec{\boldsymbol{L}})$ (with the definition $U(0) = 0$). It is given by

$$G(\vec{\boldsymbol{L}}) = \frac{1}{1 + \exp\left[-\frac{L_{\mathrm{x}}F_{\mathrm{s}} - U(\vec{\boldsymbol{L}})}{k_{\mathrm{B}}T_{\mathrm{eff}}}\right]} \tag{S10}$$

where T_{eff} is an effective temperature fit parameter. In particular, as considered in previous works [32,40], for a longitudinal backward (plus-end directed) pulling force (defining it with $F > 0$) $U(\vec{\boldsymbol{L}}) = FL_{\mathrm{x}}$ and

$$G(\vec{\boldsymbol{L}}) = \frac{1}{1 + \exp\left[-\frac{L_{\mathrm{x}}(F_{\mathrm{s}} - F)}{k_{\mathrm{B}}T_{\mathrm{eff}}}\right]} \tag{S11}$$

The above assumptions are not sufficient for the model adjustments, i.e., obtaining $W(\vec{\boldsymbol{L}})$, as for the *yeast* dynein case. For example, to the best of our knowledge, for the *mammalian* dynein case, no data is published on the inter-MTBD stretching distance distribution and/or its angular distribution. Therefore, to complete the model, we use here the very recent single-molecule results on *mammalian* dynein stepping statistics of Yildiz and co-workers [38,53]. To do so, we consider only the experimental data regarding the BICD2 adapter, as this was shown to bind mainly single dynein (~85 %) [38]. We use the experimental data for the longitudinal step-size distribution of the dynein pivot under stalling conditions, i.e. $F = F_{\mathrm{s}}$ in Eq. S11. Under such conditions, our model reads $P(\vec{\boldsymbol{L}}) = \frac{1}{2} A\, W(\vec{\boldsymbol{L}})$. Yet, the provided data [38,53]only entails the distribution of the longitudinal component L_{x}. Thus, to obtain $W(\vec{\boldsymbol{L}})$, we build on the assumption (i) described in the previous paragraph, which implies that the one-to-one correspondence between longitudinal and transverse step components (L_{x} and L_{y}, respectively) is identical for both *yeast* and *mammalian* dynein motors. Using this correspondence, we can infer the distribution of the *mammalian* L_{y} from its L_{x} distribution and, in turn, its $\vec{\boldsymbol{L}}$ distribution. In addition, to artificially reach adequate data sampling of the experimental step distribution, we build on the symmetry discussed above for $W(\vec{\boldsymbol{L}})$, which implies that ideally the raw data [38.53] should be composed of pairs of $\{\vec{\boldsymbol{L}}, -\vec{\boldsymbol{L}}\}$ abundance. We observed that in the raw data some pairs are not completed (probably due to the statistical error associated with finite-size sampling of the side steps, and not due to experimental issues). Thus, we have forced the symmetry over the given data by completing the lacking pairs.

Next, we obtained the fit parameter T_{eff} by adjusting its value such that under no external force, $F = 0$, the plus-end directed and minus-end directed steps fractions are consistent with the measured values [38]: 23 % and 77 %, respectively, leading to $T_{\mathrm{eff}} =$ 1900 K. To validate the mentioned adjustments, we compared our predictions for the mean step-size with the data [53] for different F values, see Table S4. Note the consistency of the experimental data with our single cytoplasmic *mammalian* dynein model predictions (within theoretical and experimental errors). We believe that the small mismatch, seen in Table S4, between model and experiment at $F = 0$ is likely due to an experimental error at vanishing laser trapping forces. In any case, it should have only a relatively small effect

on the motion of the NP, for the following reasons. First, during the periods with simultaneous binding of more than one motor, it is very rare to find a motor at zero load. Second, during the periods of single motor binding, the only effect of the above mismatch is on the temporal velocity, which we adjust to the known value by fitting the step time to 0.0108 s.

Table S4. Comparison between experimental and theoretical mean step-size of a single dynein (*mammalian*-type). Since the experimental results are affected by the FIONA method resolution of ± 1 nm, the error in the experimental results is estimated as $\pm\sqrt{1 + \mathrm{SEM}^2}$.

F **[pN]**	**Experimental mean step-size [nm]**	**Theoretical mean step-size [nm]**
0	11.4 ± 1.1	9.1
1.5	5.4 ± 1.5	6.9
2.3	4.3 ± 1.5	5.2
2.9	2.5 ± 1.5	3.3
3.6	-0.5 ± 1.5	0.3

6.2 Application of the single-motor stepping model to the multi-motor NP motion

The single *mammalian* dynein stepping model described above, which builds on our previous work on single *yeast* dynein stepping model and uses the experimental data of Yilditz and co-workers [38,53], can be readily applied to any of the NP motors that are attached to the MT and attempt to step. Let $U_{\mathrm{NP}}(\{\vec{\boldsymbol{r}}_{\mathbf{i}}\})$ be the NP Helmholtz free-energy for a given configuration of MT bound motors $\{\vec{\boldsymbol{r}}_{\mathbf{i}}\}$ (denoting the bound motors locations), in which the NP is in mechanical equilibrium, $\partial U_{\mathrm{NP}}(\{\vec{\boldsymbol{r}}_{\mathbf{i}}\})/\partial\vec{\boldsymbol{r}}_{\mathbf{i}} = 0$ (for all bound motors i). The zero of energy is defined, for convenience, when there are no motors bound to the MT. To apply this free-energy to Eq. S10, we assume that after a step is performed, mechanical equilibrium is instantaneously restored, as described above, and the NP coordinates are updated accordingly. Hence, we identify the "external potential" in Eq. S10 as the difference in the NP Helmholtz free-energy upon stepping of a single motor, $U(\vec{\boldsymbol{L}}) \equiv U_{\mathrm{NP}}(\{\vec{\boldsymbol{r}}_{\mathbf{i}}\}_{\mathrm{f}}) - U_{\mathrm{NP}}(\{\vec{\boldsymbol{r}}_{\mathbf{i}}\}_{\mathrm{i}})$, where $\{\vec{\boldsymbol{r}}_{\mathbf{i}}\}_{\mathrm{f}}$ and $\{\vec{\boldsymbol{r}}_{\mathbf{i}}\}_{\mathrm{i}}$ are the final and initial bound motor positions.

6.2.1. Motor free-energy

We assume that the anchoring density is in the so-called "mushroom regime" [77] (consistent with the experimental densities) such that $\xi > R_{\mathrm{g}}$. Furthermore, upon binding of a motor to the MT surface followed by stepping, the polymer becomes stretched such that the density of monomers in the transverse direction to the polymer end-to-end vector is reduced. Hence, polymer-polymer excluded volume interaction is not accounted for, and regarding the free-energies calculation, we can consider single polymer theory.

We choose a planar surface that is tangent to the spherical NP surface at the polymer anchoring position, see Figure S8. When the dynein at the polymer free-end (henceforth "dynein-polymer-end") binds to a site on the MT surface, the polymer-end can be regarded as (temporarily) fixed in space, with position dictated by the dynein and importins dimensions (see Figure S8C and Figure 2C of the main text). Thus, dynein-polymer-end position is always residing on a virtual cylindrical surface whose cross-section radius (R_2 in Figure S8C) is the sum of the MT radius, the total dynein length, and the two importin protein sizes (Supplementary Material Section 4).

To describe the free-energy change upon a binding event, we use a function that accounts for the entropy loss of a polymer upon binding, and the dynein binding energy gain, $\epsilon > 0$. Using cylindrical coordinates $(\vec{\boldsymbol{\rho}}, Z)$ associated with this tangent surface (Figures S8A,B), the polymer anchoring position is set at the origin (*0,0*), and the polymer other end, which is not anchored to the surface, is fixed at $(\vec{\boldsymbol{\rho}}, Z)$.

This leads to the following equation for the free-energy *difference*, U_{motor}, between a fixed and free-end polymer [86,87]:

$$U_{\text{motor}} = -\epsilon - k_B T \times \left[\ln\left(\frac{9Za^3}{2\pi R_0^2}\right) - \frac{3}{2}\left(\frac{\rho^2 + Z^2}{R_0^2}\right) \right] \tag{S12}$$

where a is the polymer Kuhn length (~0.76 nm for PEG [72,73]) and $R_0 = \sqrt{N_a}\, a$ is the free-polymer end-to-end distance (N_a is the number of Kuhn segments) (see Supplementary Material Section 2). We approximate ϵ to be 8 $k_B T$ [88].

Note that this free-energy approximately accounts for the excluded volume interaction between the whole polymer and the NP rigid surface (assuming $R_g \ll R$). It does not account for the excluded-volume interaction between the polymer and the MT surface. However, since the contour length of the PEG is about 40.8 nm (Supplementary Material Section 2) it is evident that no monomer can ever reach the MT itself. The excluded-volume interaction between the polymer and the body of the dynein-$\alpha\beta$ complex is neglected since – for similar considerations – the only domains where it could be relevant are the dynein tail and the two importins whose physical dimensions are relatively small.

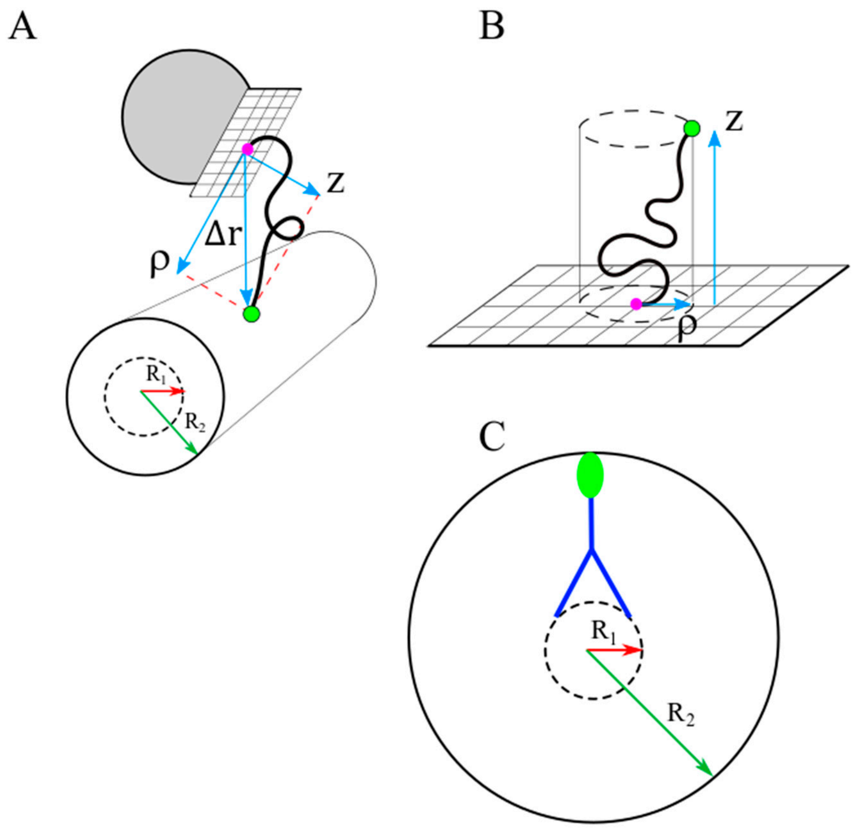

Figure S8. Illustration of the cylindrical coordinate system (ρ, z). Note that the effective radius of the cylinder, R_2 (green arrow) entails the MT radius, R_1 (red arrow), the vertical length of the dynein (blue lines), and the vertical length of the $\alpha\beta$ importin complex (green ellipse). Therefore, the value of R_2 is taken as 60 nm (the length estimation is discussed in detail in Supplementary Material Section 4). **(A)** Illustration of the coordinate system for the case of a polymer that connects between a NP (gray circle) and the $\alpha\beta$ importin complex (green dot) that in turn, is connected to the dynein tail domain. **(B)** Illustration of the coordinates for the general case. **(C)** Illustration of the cylindrical cross-sections of (A).

As each polymer in anchored at a different position on the NP surface, its anchoring tangent plane is orientated differently with respect to the MT surface. Thus, we perform a coordinate transformation for each polymer to obtain its specific U_{motor}. In Eq. S12, $\rho^2 + Z^2$ stands for the square of the (actual) end-to-end vector $\Delta\vec{r}$ (Figures S8A,B), which we calculate for each MT bound motor. This can be conveniently done by transforming to lab frame Cartesian coordinate system, redefining the origin at the NP center, and using $Z =$

$\hat{\boldsymbol{n}} \cdot \Delta\vec{\boldsymbol{r}}$ where $\hat{n}$ the unit vector normal to the NP surface at the anchoring position (pink dot, Figures S8A,B).

6.2.2. Motor Stepping rate

For a single free motor, the mean stepping rate for any step-size $\vec{\boldsymbol{L}}$, k_{step}, can be estimated from $k_{\mathrm{step}} = \langle v_{\mathrm{x}}\rangle/\langle L_{\mathrm{x}}\rangle$, where v_{x} is the longitudinal velocity [nm/s], and L_{x} is the longitudinal step-size [nm]. For *mammalian* cytoplasmic dynein, we set $\langle v_{\mathrm{x}}\rangle$ to be the well-established value 800 nm/s and $\langle L_{\mathrm{x}}\rangle$ to be about 9 nm. This implies that the step-size dependent stepping rate is $k_{\mathrm{step}}(\vec{\boldsymbol{L}}) = k_{\mathrm{step}}P(\vec{\boldsymbol{L}})$, where $P(\vec{\boldsymbol{L}})$ is the *normalized* step-size distribution defined in Eq. S9. Thus, consistently, $\langle v_{\mathrm{x}}\rangle = \int_0^{\infty} \mathrm{d}L_{\mathrm{x}}\, k_{\mathrm{step}}(L_{\mathrm{x}})\, L_{\mathrm{x}} = k_{\mathrm{step}}\langle L_{\mathrm{x}}\rangle$. Note that k_{step} includes the dwell time.

6.2.3. Motor Binding-Unbinding rates

As described in Ref. [16], we assume that the binding-unbinding rates of the different motors obey detailed balance relations. Therefore, the pair of rates, k_+ – for binding of a single motor, and k_- – for the unbinding of the same motor, are assumed to obey the ratio $\frac{k_+}{k_-} = \exp\left[-\frac{U_{\mathrm{NP},+} - U_{\mathrm{NP},-}}{\mathrm{k_B T}}\right]$, where $U_{\mathrm{NP},+} \equiv U_{\mathrm{NP}}(\{\vec{\boldsymbol{r}}_{\mathrm{i}}\}_+)$ and $U_{\mathrm{NP},-} \equiv U_{\mathrm{NP}}(\{\vec{\boldsymbol{r}}_{\mathrm{i}}\}_-)$ are the free-energies of the binding and unbinding states, respectively. In order to uniquely specify the two states, there is (as usual) a need for another condition, and (consistent with common choices in stochastic processes) we take $k_+ + k_- = \tau^{-1}$, and adjust the free-parameter τ^{-1} such that when the NP is left with a single motor it unbinds with the experimentally known rate $k_0 = 1$ Hz [32]. As shown in Ref. [16], this leads to the following rate expressions:

$$k_- = \tau^{-1} \frac{1}{1 + \exp\left(\frac{\Delta U_{\mathrm{NP}}}{\mathrm{k_B T}}\right)} \tag{S13}$$

$$k_+ = \tau^{-1} \frac{\exp\left(\frac{\Delta U_{\mathrm{NP}}}{\mathrm{k_B T}}\right)}{1 + \exp\left(\frac{\Delta U_{\mathrm{NP}}}{\mathrm{k_B T}}\right)} \tag{S14}$$

$$\tau^{-1} = k_0 \left(1 + \exp\left(\frac{\Delta U_0}{\mathrm{k_B T}}\right)\right) \tag{S15}$$

where $\Delta U_{\mathrm{NP}} = U_{\mathrm{NP},+} - U_{\mathrm{NP},-}$ is the NP free-energy difference between a (single motor) binding and unbinding states, and ΔU_0 is associated with the special case where the NP entails only a single motor. Note again that ΔU_{NP} accounts for the free-energy change of the whole NP (upon a single binding event) of any one of the motors out of all the NP anchored motors, and when both states are assumed to be at mechanical equilibrium.

6.2.4. Motor-motor excluded volume interaction

When a motor attempts to step into a binding site, it may not be accessible due to the "excluded volume interaction" between the attempting motor and the neighboring bound motors and we term this as a "jamming event". If a jamming event occurs, the attempting motor rests still, implying that the temporal NP longitudinal and angular velocities (v_{x} and ω) both vanish. We determine the motor-motor excluded volume interaction using the following approach. First, the volume occupied by a single MTBD is described as that of a sphere of 4 nm radius [64] (Figure S9), which allows determining the excluded volume created when two MTBDs meet, i.e. the volume of a sphere of the MTBD diameter. Note that the known MTBD measured dimensions, 3.6 nm, are likely smaller than those we use; this practice ensures that we do not underestimate the volume occupied by the MTBD.

Second, let us now define $V_{\mathrm{pair},\langle ij\rangle}$ as the volume occupied by the $\langle ij\rangle$ MTBD *pair*, where the space between the MTBDs is also accounted (Figure S9A). Thus, a jamming event occurs whenever the volumes of two pairs (corresponding to two motors), $V_{\mathrm{pair},\langle ij\rangle}$ and $V_{\mathrm{pair},\langle km\rangle}$ ($\langle ij\rangle \neq \langle km\rangle$), overlap. However, since our model does not consider the actual dynein structure, we determined the average distance between two motors that leads to a jamming event, d_{jam} (Figures S9C–E). We represent each pair as a projection on a 2D surface, or in other words as a ribbon of length ℓ, capped with two hemi-circles (Figure S9B).

Next, we estimate $\langle d_{\mathrm{jam}}\rangle$ using the following calculation (Figures S9C,D). We consider two such ribbons at contact (Figures S9F), making an angle α between them. The vector positions of the two ribbon centers obey, $|\vec{\boldsymbol{r_1}}| = \frac{\ell}{2} - x$, $|\vec{\boldsymbol{r_2}}| = \frac{\ell}{2}$. The vector connecting the two ribbon centers is $\vec{\boldsymbol{u}} = \vec{\boldsymbol{r_2}} - \vec{\boldsymbol{r_1}}$. Therefore $u^2 = r_2^2 + r_1^2 - 2r_2r_1\cos\alpha$, leading to $u^2 = \left(\frac{\ell}{2}\right)^2 + \left(\frac{\ell}{2} - x\right)^2 - 2\left(\frac{\ell}{2}\right)\left(\frac{\ell}{2} - x\right)\cos\alpha$. The configurational average of this distance is $\langle u\rangle_{\mathrm{config}} = \frac{2}{\pi}\int_0^{\frac{\pi}{2}} \mathrm{d}\alpha \frac{1}{\ell}\int_0^{\ell} \mathrm{d}x\, u(x,\alpha)$, which can be evaluated numerically to give $\langle u\rangle_{\mathrm{config}} \cong 0.543\,\ell$. As the value of ℓ can vary, we use its mean, namely $\langle d_{\mathrm{jam}}\rangle = 0.543\,\langle\ell\rangle = 12$ nm.

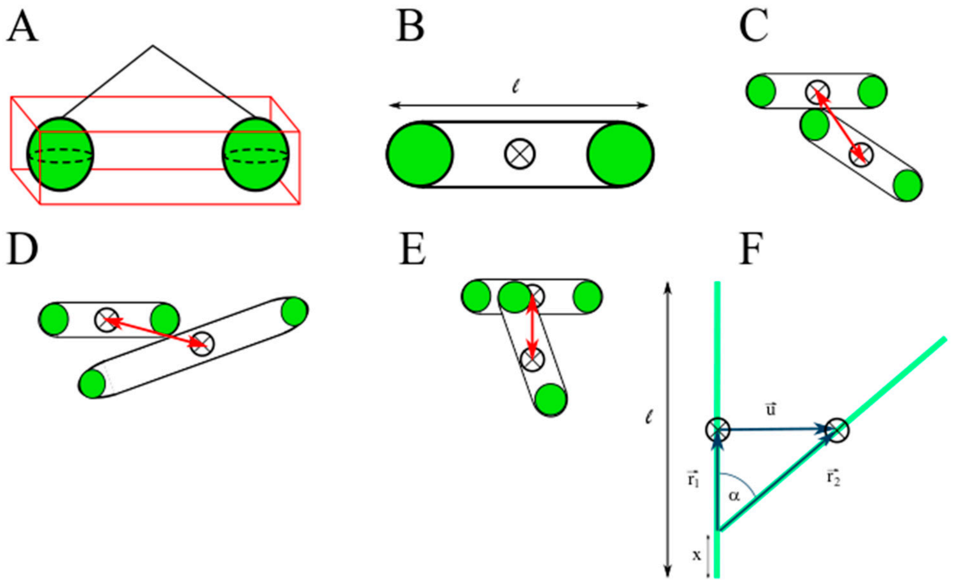

Figure S9. Illustration of the excluded volume and possible configurations of two MTBD pairs. MTBDs are represented by green spheres. The dynein pivots are represented by ⊗, and the distance between two dynein pivots (d_{jam}) is represented by the red arrows. **(A)** Illustration of the volume occupied by a MTBD pair and the space in between the pair (red square). **(B)** Illustration of the projection of (A). **(C-D)** Two examples of two adjacent MTBD pairs. **(E)** Example of two MTBD pairs in "crossing" configuration. **(F)** Schematic illustration of a possible configuration of two MTBD pairs. MTBD pairs are represented by green lines (of length ℓ), and the dynein pivots are represented by ⊗.

Note that this approximation does not account for crossing configurations (Figure S9E). Thus, $\langle d_{\mathrm{jam}}\rangle = 12$ nm can only be taken as an upper bound value estimate. To estimate a lower bound value, we need to account for crossing configurations. However, as far as we know, there is no data concerning dynein-dynein crossing configurations, so we only examine a simple crossing case in which the likelihood for steric interferences is relatively low; we consider the case where one MTBD resides between another pair of MTBDs belonging to a neighboring motor (Figure S9E). We use the same method as above, but without accounting for the MTBDs dimension, resulting in shorter ribbon length, $\ell^* = \ell - 2d_{\mathrm{MTBD}}$ (where $d_{\mathrm{MTBD}} = 4$ nm is the "MTBD sphere" radius) such that $\langle d^*_{\mathrm{jam}}\rangle \cong 7.7$ nm. Since the real value of d_{jam} resides between the lower and the upper estimated bounds (i.e., between 7.7 and 12 nm), we use in our simulations $\langle d_{\mathrm{jam}}\rangle = 10$ nm. Consequently, regarding the excluded volume, our motors are modeled as standing cylinders,

with a cross-section diameter of 10 nm and vertical length as estimated in Supplementary Material Section 4.

6.2.5. Time scales of fast, non-simulated, processes

There are a few microscopic dynamical processes associated with the NP dynamics that were not accounted for explicitly in our model. As shown below, these processes are much faster than both timescales for motor stepping and motor binding-unbinding kinetics. This separation of timescales allows us to use a few model assumptions mentioned above and in Section 3.3 of the main text.

Consider first the Zimm relaxation time for the spacer polymer configurational fluctuations, $\tau_Z \approx \frac{\eta R_g^3}{k_B T}$, where η is the medium viscosity, and R_g is the gyration radius. More precisely, for a Gaussian chain [70], $\tau_Z = 0.325 \frac{\eta R_0^3}{k_B T} \simeq$ 12 ns, for the value of $R_0 \simeq 5.56$ nm used in this work (see Supplementary Material Section 2). This is about 5 orders of magnitude shorter than the shortest MC step time in our simulations ($\tau_{MC} \sim 1$ ms). Hence, the polymer may be assumed to explore all its configurational phase space during any stepping, binding, or unbinding event (henceforth "MC-event"), which justifies the use of a polymer free-energy without accounting for the polymer dynamics explicitly.

Next, we consider the NP translational and rotational motion. They are involved in the mechanical re-equilibration of the mean NP center-of-mass position and orientation after an MC-event. They are also involved in the relaxation of thermal fluctuations around the mean position when no such event takes place. For the translational re-equilibration motion, assume that, after an MC-event, the NP translates a distance r to reach translational mechanical equilibrium. This generates a (polymer) restoring force of magnitude $f = K_p r$, where K_p is the entropic (free-polymer) spring constant, $K_p = 3k_B T/R_0^2$. Note that – for large extensions (i.e., when $r \gg R_0$) – it is much higher, therefore this force can serve as an estimated lower bound. Using the NP translational Stokes drag coefficient, $\gamma_t = 6\pi\eta R$, we find an (upper bound) estimate for the translational relaxation time, $\tau_{trans} = \frac{\gamma_t}{K_p} = \frac{2\pi\eta R R_0^2}{k_B T} \simeq 8.4 \times 10^{-7}$ s (taking the NP radius $R \simeq 20$ nm). This value is about three orders of magnitude shorter than the shortest MC step time in our simulations, $\tau_{MC} \sim 1$ ms, thus allowing to assume that the translational equilibration occurs instantaneously after each MC-event.

Next, we wish to estimate the order of magnitude of the NP rotational equilibration time, avoiding exhaustive details associated with the geometry. Assume that, after an MC-event, the NP requires to rotate an angle θ to reach rotational mechanical equilibrium. The torque T exerted by a force f on the NP is roughly $T \sim fR \simeq K_p r R$, and the angle θ is related to the associated polymer extension by roughly $r \sim R\,\theta$ leading to a (restoring) torque $T \sim K_p R^2 \theta$. Using the rotational Stokes drag coefficient of the NP, $\gamma_\theta = 8\pi\eta R^3$, leads to the following estimate for the rotational relaxation time, $\tau_{rot} \sim \frac{\gamma_\theta}{K_p R^2} \simeq \left(\frac{8\pi}{3}\right)\frac{\eta R R_0^2}{k_B T} \sim \tau_{trans} \ll \tau_{MC}$, leading to the same conclusion.

Linear response theory suggests that the same relaxation times also govern the relaxation of thermal fluctuations near equilibrium, again suggesting that it is adequate to consider the mean center-of-mass position and mean orientation of the NP before and after an MC-event.

7. Simulations Results for $\Delta t = 0.27$ s

Table S5. Data for Figure 7A. Mean longitudinal velocity, $\langle v_x \rangle$ (mean $\pm$ SEM) and STD, $\langle \delta v_x \rangle$, of the different ($R =$ 20 nm, N_m) configurations for time intervals equal to 0.27 s.

N_m	1	2	3	4	5	6	7
$\langle v_x \rangle$	833 ± 4	769 ± 3	693 ± 2	640 ± 2	605 ± 1	584 ± 1	569 ± 1
$\langle \delta v_x \rangle$	245	297	322	326	321	315	308
N_m	8	9	10	11	12	13	
$\langle v_x \rangle$	557 ± 1	547 ± 1	540 ± 1	533 ± 1	528 ± 1	523 ± 1	
$\langle \delta v_x \rangle$	302	296	291	286	282	277	

Table S6. Data for Figure 7C. Mean longitudinal velocity $\langle v_x \rangle$ (mean $\pm$ SEM) separated for minus-end directed, $v_x > 0$, and plus-end directed, $v_x < 0$, directions for the different ($R = 20$ nm, N_m) configurations for time intervals of 0.27 s.

N_m	1	2	3	4	5	6	7
$\langle v_x \rangle \mid v_x > 0$	833 ± 4	776 ± 3	705 ± 2	654 ± 2	621 ± 1	600 ± 1	584 ± 1
$\langle v_x \rangle \mid v_x < 0$	N/A	-38 ± 4	-37 ± 2	-38 ± 1	-38 ± 1	-39 ± 1	-39 ± 1
N_m	8	9	10	11	12	13	
$\langle v_x \rangle \mid v_x > 0$	572 ± 1	562 ± 1	555 ± 1	548 ± 1	542 ± 1	537 ± 1	
$\langle v_x \rangle \mid v_x < 0$	-39 ± 1	-40 ± 1	-40 ± 1	-40 ± 1	-40 ± 1	-40 ± 1	

Table S7. Data for Figure 9A. Mean angular velocity $\langle \omega \rangle$ (mean $\pm$ SEM) and STD, $\langle \delta\omega \rangle$, of the different ($R = 20$ nm, N_m) configurations for time intervals equal 0.27 s.

N_m	1	2	3	4	5	6	7
$\langle \omega \rangle$	1.97 ± 0.14	1.79 ± 0.09	1.47 ± 0.06	1.28 ± 0.04	1.10 ± 0.03	0.97 ± 0.02	0.87 ± 0.02
$\langle \delta\omega \rangle$	8.16	7.88	7.28	6.78	6.37	6.06	5.80
N_m	8	9	10	11	12	13	
$\langle \omega \rangle$	0.80 ± 0.01	0.74 ± 0.01	0.68 ± 0.01	0.63 ± 0.01	0.59 ± 0.01	0.56 ± 0.01	
$\langle \delta\omega \rangle$	5.58	5.38	5.21	5.05	4.91	4.78	

Table S8. Data for Figure 9C. Mean angular velocity, $\langle \omega \rangle$, separated for minus-end directed, $v_x > 0$, and plus-end directed, $v_x < 0$, motions for the different ($R = 20$ nm, N_m) configurations for time intervals of 0.27 s. Values correspond to (mean $\pm$ SEM).

N_m	1	2	3	4	5	6	7
$\langle \omega \rangle \mid v_x > 0$	1.97 ± 0.14	1.81 ± 0.09	1.50 ± 0.06	1.31 ± 0.04	1.12 ± 0.03	0.99 ± 0.02	0.90 ± 0.02
$\langle \omega \rangle \mid v_x < 0$	N/A	0.05 ± 0.24	0.05 ± 0.12	0.05 ± 0.08	0.04 ± 0.06	0.06 ± 0.04	0.07 ± 0.03
N_m	8	9	10	11	12	13	
$\langle \omega \rangle \mid v_x > 0$	0.82 ± 0.01	0.75 ± 0.01	0.69 ± 0.01	0.65 ± 0.01	0.61 ± 0.01	0.57 ± 0.01	
$\langle \omega \rangle \mid v_x < 0$	0.07 ± 0.03	0.06 ± 0.02	0.06 ± 0.02	0.06 ± 0.02	0.06 ± 0.02	0.06 ± 0.01	

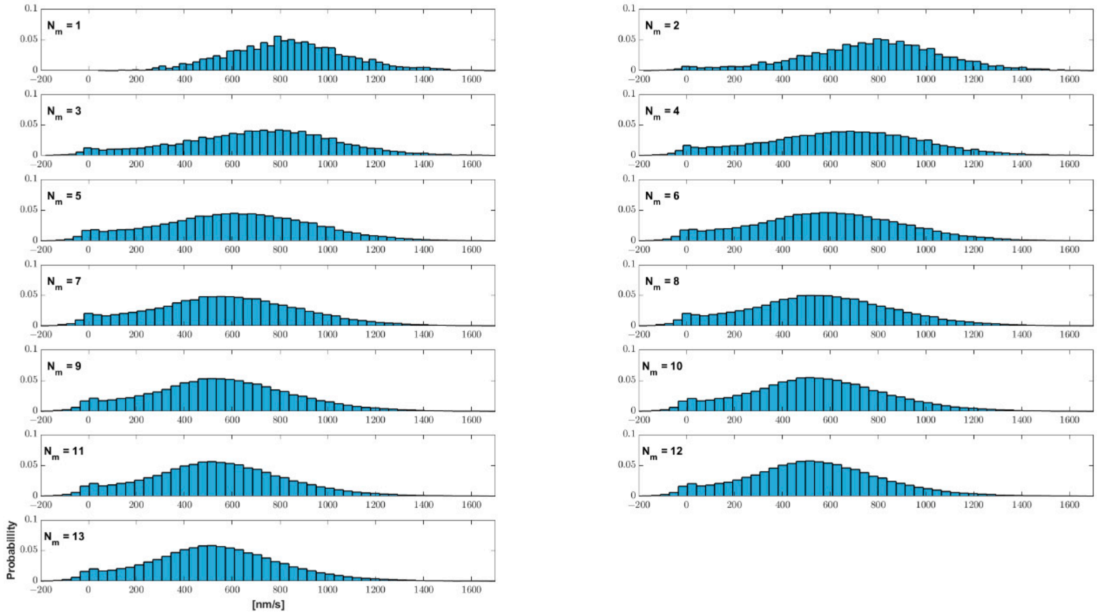

Figure S10. Distributions of the longitudinal velocity, υ_x, for the different ($R = 20$ nm, N_m) configurations for time interval of 0.27 s.

8. Simulation Results for Δt = MC time step

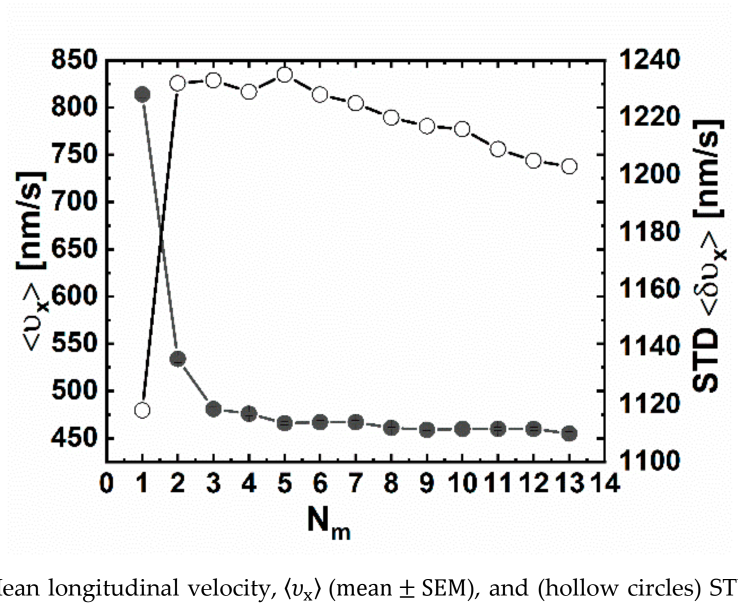

Figure S11. (Black dots) Mean longitudinal velocity, $\langle \upsilon_x \rangle$ (mean $\pm$ SEM), and (hollow circles) STD, $\langle \delta\upsilon_x \rangle$, of the different ($R = 20$ nm, N_m) configurations for time intervals equal to MC step times.

Table S9. Data for Figure S11. Mean longitudinal velocity, $\langle \upsilon_x \rangle$ (mean $\pm$ SEM) and STD, $\langle \delta\upsilon_x \rangle$, of the different ($R =$ 20 nm, N_m) configurations for time intervals equal to MC step times.

N_m	**1**	**2**	**3**	**4**	**5**	**6**	**7**
$\langle \upsilon_x \rangle$	814 ± 4	534 ± 4	481 ± 2	458 ± 2	466 ± 2	467 ± 2	467 ± 2
$\langle \delta\upsilon_x \rangle$	1188	1232	1233	1227	1235	1228	1225
N_m	**8**	**9**	**10**	**11**	**12**	**13**	
$\langle \upsilon_x \rangle$	461 ± 1	459 ± 1	460 ± 1	454 ± 2	451 ± 1	452 ± 1	
$\langle \delta\upsilon_x \rangle$	1220	1217	1216	1208	1203	1202	

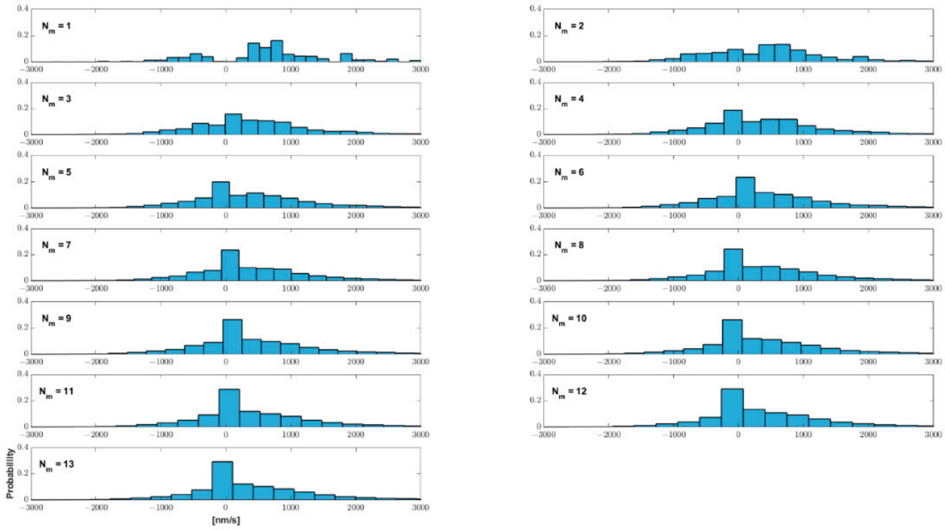

Figure S12. Distributions of the longitudinal velocity, v_x, for the different ($R = 20$ nm, N_m) configurations for time intervals equal to MC step times.

Table S10. Data for Figure 7B. Mean longitudinal run-lengths (along the MT symmetry axis), $\langle \lambda \rangle$, and run-times, $\langle \tau_p \rangle$, for the different ($R = 20$ nm, N_m) configurations for time intervals equal to MC step times. These values are effectively unaffected if the detection of the NP detachment events (i.e., the last motor unbinding event) takes place on a time interval of 0.27 s, (introducing larger errors in these values). Values correspond to (mean ± SEM).

$\boldsymbol{N_m}$	**1**	**2**	**3**	**4**	**5**	**6**	**7**
$\langle \lambda \rangle$	823 ± 27	798 ± 27	935 ± 33	1132 ± 40	1474 ± 52	1528 ± 50	1677 ± 56
$\langle \tau_p \rangle$	1 ± 0.03	1.16 ± 0.04	1.65 ± 0.06	2.14 ± 0.08	2.82 ± 0.1	2.95 ± 0.1	3.28 ± 0.11
$\boldsymbol{N_m}$	**8**	**9**	**10**	**11**	**12**	**13**	
$\langle \lambda \rangle$	2037 ± 69	2119 ± 71	2434 ± 79	2592 ± 87	2797 ± 91	3123 ± 102	
$\langle \tau_p \rangle$	4.07 ± 0.14	4.28 ± 0.14	4.93 ± 0.16	5.33 ± 0.18	5.81 ± 0.19	6.49 ± 0.21	

Table S11. Data for Figure 8A. Mean number and STD of transient MT-bound motors, M_B, for different ($R = 20$ nm, N_m) configurations for time intervals equal to MC step times.

$\boldsymbol{N_m}$	**1**	**2**	**3**	**4**	**5**	**6**	**7**
$\langle M_B \rangle$	1	1.38	1.63	1.73	1.81	1.85	1.94
$\langle \delta M_B \rangle$	0	0.4883	0.5142	0.5123	0.5075	0.5110	0.5107
$\boldsymbol{N_m}$	**8**	**9**	**10**	**11**	**12**	**13**	
$\langle M_B \rangle$	1.94	1.97	2.01	2.04	2.07	2.09	
$\langle \delta M_B \rangle$	0.5174	0.5280	0.5363	0.5459	0.5590	0.5638	

Table S12. Data for Figure 8B. Time fraction of the different NP states, where a state is defined according to the number of MT bound motors, M_B, for different ($R = 20$ nm, N_m) configurations and for time intervals equal to MC step times.

$\boldsymbol{N_m}$ / State	**1**	**2**	**3**	**4**	**5**	**6**	**7**
$M_B = 1$	1	0.83	0.53	0.44	0.34	0.29	0.26
$M_B = 2$	0	0.17	0.46	0.54	0.62	0.65	0.67
$M_B = 3$	0	0	0.01	0.02	0.04	0.06	0.07
$M_B = 4$	0	0	0	< 0.01	< 0.01	< 0.01	< 0.01
$M_B = 5$	0	0	0	0	< 0.01	< 0.01	< 0.01
	**8**	**9**	**10**	**11**	**12**	**13**	
$M_B = 1$	0.23	0.21	0.18	0.17	0.15	0.14	
$M_B = 2$	0.69	0.70	0.71	0.72	0.72	0.72	
$M_B = 3$	0.08	0.09	0.10	0.10	0.12	0.13	
$M_B = 4$	< 0.01	< 0.01	0.01	0.01	0.01	0.01	

Table S13. Data for Figure 8C. Mean longitudinal velocity of the different NP states, where a state is defined according to the number of MT bound motors, M_B, for the different ($R = 20$ nm, N_m) configurations for time intervals equal to MC step times (mean ± SEM).

N_m	1	2	3	4	5	6	7
$\langle v_{x,M_B=1}\rangle$	813 ± 6	819 ± 9	826 ± 10	808 ± 8	822 ± 9	819 ± 9	811 ± 8
$\langle v_{x,M_B=2}\rangle$	N/A	154 ± 10	286 ± 8	358 ± 7	395 ± 6	409 ± 5	428 ± 5
$\langle v_{x,M_B=3}\rangle$	N/A	N/A	148 ± 58	168 ± 18	185 ± 9	204 ± 8	219 ± 7
$\langle v_{x,M_B=4}\rangle$	N/A	N/A	N/A	75 ± 30	142 ± 37	101 ± 25	143 ± 28
$\langle v_{x,M_B=5}\rangle$	N/A	N/A	N/A	N/A	0	0	0
N_m	8	9	10	11	12	13	
$\langle v_{x,M_B=1}\rangle$	818 ± 9	812 ± 8	821 ± 8	804 ± 7	818 ± 8	814 ± 9	
$\langle v_{x,M_B=2}\rangle$	445 ± 6	450 ± 5	456 ± 4	457 ± 4	460 ± 4	466 ± 4	
$\langle v_{x,M_B=3}\rangle$	236 ± 7	244 ± 6	241 ± 6	248 ± 5	242 ± 4	255 ± 5	
$\langle v_{x,M_B=4}\rangle$	126 ± 19	108 ± 13	114 ± 12	148 ± 14	128 ± 9	129 ± 7	
$\langle v_{x,M_B=5}\rangle$	0	-37 ± 15	54 ± 51	6 ± 26	59 ± 18	60 ± 16	

Table S14. Longitudinal velocity STD of the different NP states. A state is defined according to the number of MT bound motors, M_B, for the different ($R = 20$ nm, N_m) configurations for time intervals equal to MC step times (see Table S13 and Figure 8C in main text for the mean velocities).

N_M	1	2	3	4	5	6	7
$\langle \delta v_{x,M_B=1}\rangle$	0	296	321	251	284	252	256
$\langle \delta v_{x,M_B=2}\rangle$	N/A	163	227	205	188	169	158
$\langle \delta v_{x,M_B=3}\rangle$	N/A	N/A	487	239	206	211	201
$\langle \delta v_{x,M_B=4}\rangle$	N/A	N/A	N/A	60	150	157	255
$\langle \delta v_{x,M_B=5}\rangle$	N/A	N/A	N/A	N/A	N/A	N/A	N/A
N_M	8	9	10	11	12	13	
$\langle \delta v_{x,M_B=1}\rangle$	285	247	254	234	256	293	
$\langle \delta v_{x,M_B=2}\rangle$	178	144	121	123	120	121	
$\langle \delta v_{x,M_B=3}\rangle$	214	179	188	145	123	157	
$\langle \delta v_{x,M_B=4}\rangle$	217	198	226	189	177	169	
$\langle \delta v_{x,M_B=5}\rangle$	N/A	N/A	141	58	80	96	

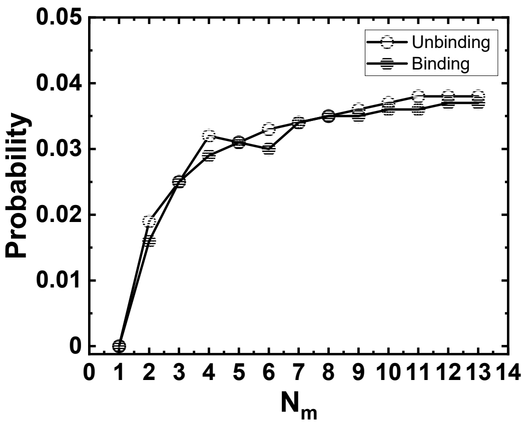

Figure S13. Fraction of binding and unbinding events out of the total possible events for the different ($R = 20$ nm, N_m) configurations for time intervals equal to MC step times.

Table S15. Data for Figure S13. Fraction of binding and unbinding events out of the total possible events for the different (R = 20 nm, N_{m}) configurations for time intervals equal to MC step times.

N_{m}	**1**	**2**	**3**	**4**	**5**	**6**	**7**
$F_{\mathrm{unbinding}}$	<0.01	0.019	0.025	0.032	0.031	0.033	0.034
F_{binding}	<0.01	0.016	0.025	0.029	0.031	0.03	0.034
N_{m}	**8**	**9**	**10**	**11**	**12**	**13**	
$F_{\mathrm{unbinding}}$	0.035	0.036	0.037	0.038	0.038	0.038	
F_{binding}	0.035	0.035	0.036	0.036	0.037	0.037	

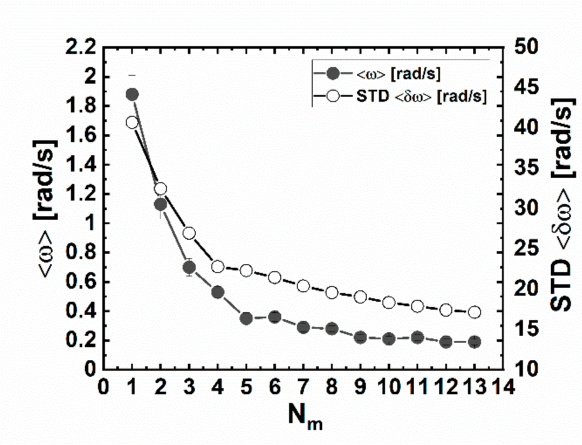

Figure S14. Mean angular velocity, $\langle\omega\rangle$ (mean ± SEM), and STD, $\langle\delta\omega\rangle$, of the different ($R$ = 20 nm, N_{m}) configurations for time intervals equal to MC step times.

Table S16. Data for Figure S14. Mean angular velocity, $\langle\omega\rangle$ (mean ± SEM), and STD, $\langle\delta\omega\rangle$, of the different ($R$ = 20 nm, N_{m}) configurations for time intervals equal to MC step times.

N_m	**1**	**2**	**3**	**4**	**5**	**6**	**7**
$\langle\omega\rangle$	1.88 ± 0.13	1.13 ± 0.1	0.70 ± 0.06	0.53 ± 0.04	0.35 ± 0.04	0.36 ± 0.03	0.29 ± 0.03
$\langle\delta\omega\rangle$	40.69	32.47	26.95	22.78	22.31	21.45	20.39
N_{m}	**8**	**9**	**10**	**11**	**12**	**13**	
$\langle\omega\rangle$	0.28 ± 0.02	0.22 ± 0.02	0.21 ± 0.02	0.22 ± 0.02	0.19 ± 0.02	0.19 ± 0.02	
$\langle\delta\omega\rangle$	19.58	19.04	18.33	17.88	17.40	17.14	

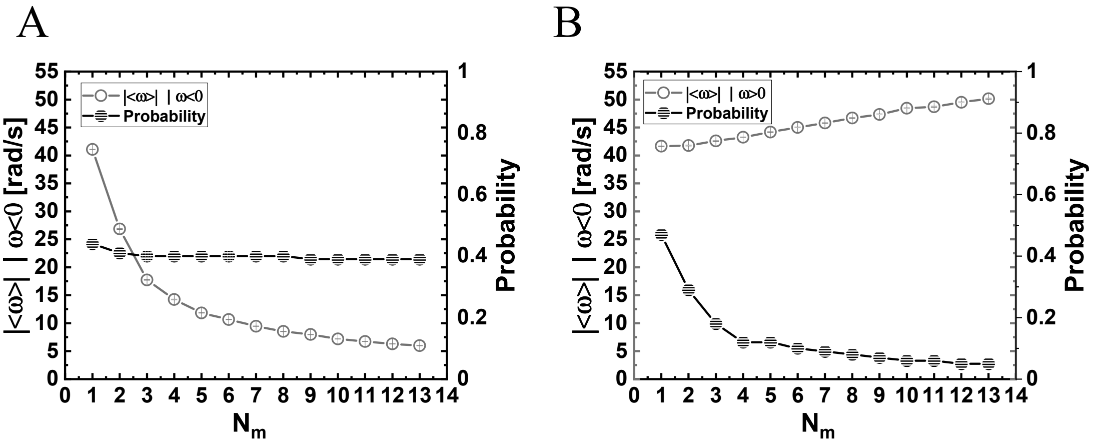

Figure S15. Mean absolute angular velocity segregated to left-handed and right-handed components of the distin*ct* (R = 20 nm, N_{m}) configurations for time intervals equal to MC step times. (A) Mean absolute left-handed angular velocity, $|\langle\omega_{\mathrm{L}}\rangle| = |\langle\omega\rangle||\ \omega < 0$, and probability for left-handed motion. (B) Mean absolute right-handed angular velocity, $|\langle\omega_{\mathrm{R}}\rangle| = |\langle\omega\rangle||\ \omega > 0$, and probability for right-handed motion.

Table S17. Data for Figure S14. Mean angular velocity separated to left- ($\langle\omega_L\rangle$) and right- ($\langle\omega_R\rangle$) handed components of the distinct ($R = 20$ nm, N_m) configurations for time intervals equal to MC step times. Values correspond to (mean ± SEM).

N_m	1	2	3	4	5	6	7
$\langle\omega_L\rangle$	-41.07 ± 0.05	-26.86 ± 0.1	-17.74 ± 0.07	-14.24 ± 0.06	-11.83 ± 0.05	-10.67 ± 0.05	-9.46 ± 0.04
$\langle\omega_R\rangle$	41.66 ± 0.05	41.76 ± 0.08	42.59 ± 0.07	43.25 ± 0.07	44.18 ± 0.07	45.02 ± 0.08	45.79 ± 0.08
N_m	8	9	10	11	12	13	
$\langle\omega_L\rangle$	-8.52 ± 0.04	-7.97 ± 0.04	-7.18 ± 0.03	-6.71 ± 0.03	-6.29 ± 0.03	-5.99 ± 0.03	
$\langle\omega_R\rangle$	46.7 ± 0.08	47.34 ± 0.09	48.44 ± 0.09	48.70 ± 0.09	49.50 ± 0.09	50.15 ± 0.09	

Table S18. Data for Figure 9B. Absolute mean angular velocity segregated to minus-end directed, $\langle\omega\rangle|\, v_x > 0$, and plus-end directed, $\langle\omega\rangle|\, v_x < 0$, motions for the distinct ($R = 20$ nm, N_m) configurations for time intervals equal to MC step times (mean ± SEM).

N_m	1	2	3	4	5	6	7
$\langle\omega\rangle \mid v_x > 0$	0.6 ± 0.15	0.5 ± 0.14	0.3 ± 0.08	0.024 ± 0.06	0.03 ± 0.05	0.14 ± 0.04	0.07 ± 0.04
$\langle\omega\rangle \mid v_x < 0$	6.8 ± 0.29	2.6 ± 0.15	1.64 ± 0.08	1.3 ± 0.07	1.16 ± 0.05	0.99 ± 0.05	0.92 ± 0.05
N_m	8	9	10	11	12	13	
$\langle\omega\rangle \mid v_x > 0$	0.09 ± 0.03	0.005 ± 0.03	0.07 ± 0.03	0.09 ± 0.03	0.06 ± 0.03	0.08 ± 0.02	
$\langle\omega\rangle \mid v_x < 0$	0.83 ± 0.04	0.8 ± 0.04	0.66 ± 0.04	0.65 ± 0.03	0.60 ± 0.03	0.56 ± 0.03	

Table S19. Data for Figure 10A. Analysis of the actual NP helical motion from observed (simulated) trajectories separated to left- and right- handed motions (mean ± SEM). $\langle H_L\rangle$ is the mean helical pitch size for left-handed helices (i.e., $\phi = -2\pi$), and $\langle H_R\rangle$ is the mean helical pitch size for right-handed helices (i.e., $\phi = +2\pi$).

N_m	1	2	3	4	5	6	7
$\langle H_L\rangle$	993 ± 97	857 ± 79	1201 ± 106	1185 ± 72	1383 ± 96	1794 ± 133	1746 ± 173
$\langle H_R\rangle$	789 ± 28	824 ± 44	992 ± 42	1173 ± 55	1542 ± 65	1565 ± 70	1671 ± 71
N_m	8	9	10	11	12	13	
$\langle H_L\rangle$	2173 ± 169	2034 ± 183	2383 ± 239	2541 ± 191	2825 ± 237	3169 ± 245	
$\langle H_R\rangle$	2026 ± 92	2151 ± 89	2575 ± 120	2602 ± 119	2780 ± 133	3068 ± 134	

Table S20. Data for Figure 10B. Analysis of the actual NP helical motion from observed (simulated) trajectories, where $\langle H\rangle$ is the mean helical pitch size (mean ± SEM), $\langle\delta H\rangle$ is the helical pitch STD, F_H is the fraction of NP trajectories that completed at least one wind around the MT symmetry axis out of the total number of trajectories, P_{LH} is the probability for a left-handed helical motion, and P_{RH} is the probability for a right-handed helical motion. The mean pitch size is calculated by averaging over all the helical pitches of the same (R, N_m) configuration, regardless of their direction (i.e., $\phi = +2\pi$ or $\phi = -2\pi$). A helical pitch size equals to the NP accumulated longitudinal motion – towards the MT minus-end – up to the point when a wind around the MT is completed (i.e., $\phi = \pm 2\pi$). Note that the motion does not have to be consistent towards either left-handed or right-handed direction, e.g., a right-handed helix can contain left-handed motion ($\Delta\phi < 0$) and vice versa, or in other words, the large transverse fluctuations do not reset the $\phi = \sum \Delta\phi$ count towards a wind completion ($\pm 2\pi$).

N_m	1	2	3	4	5	6	7
$\langle H\rangle$	825 ± 29	832 ± 38	1032 ± 40	1176 ± 46	1503 ± 55	1619 ± 62	1689 ± 68
$\langle\delta H\rangle$	548	570	695	815	1041	1092	1228
F_H	0.25	0.23	0.22	0.22	0.27	0.24	0.24
P_{LH}	0.18	0.24	0.2	0.2	0.25	0.24	0.24
P_{RH}	0.82	0.76	0.80	0.8	0.75	0.76	0.76
N_m	8	9	10	11	12	13	
$\langle H\rangle$	2061 ± 81	2124 ± 80	2533 ± 108	2588 ± 102	2792 ± 116	3091 ± 118	
$\langle\delta H\rangle$	1565	1470	1987	1956	2152	2320	
F_H	0.28	0.26	0.25	0.27	0.25	0.28	
P_{LH}	0.24	0.23	0.22	0.22	0.26	0.23	
P_{RH}	0.76	0.77	0.78	0.78	0.64	0.77	

Table S21. Data for Figure 10C. Helical pitch size estimated from the simulation results for the angular and longitudinal mean velocity values, $\langle \bar{H} \rangle = |\frac{2\pi}{\langle \omega \rangle} \langle v_x \rangle|$, together with the observed pitch size, $\langle H \rangle$, i.e., the one obtained directly from the simulations. Values correspond to (mean ± SEM).

N_m	1	2	3	4	5	6	7
$\langle H \rangle$	825 ± 29	832 ± 38	1032 ± 40	1176 ± 46	1503 ± 55	1619 ± 62	1689 ± 68
$\langle \bar{H} \rangle$	2718 ± 192	2965 ± 265	4344 ± 278	5383 ± 321	8299 ± 544	8035 ± 467	9936 ± 597
N_m	8	9	10	11	12	13	
$\langle H \rangle$	2061 ± 81	2124 ± 80	2533 ± 108	2588 ± 102	2792 ± 116	3091 ± 118	
$\langle \bar{H} \rangle$	10298 ± 554	13045 ± 812	13585 ± 837	12699 ± 696	14967 ± 856	15208 ± 837	

9. Binomial Distribution of NP Bound Motors

The number of motors N_m that are bound to the NP (not to be confused with the MT-bound motors) is a random variable, as in any adsorption process to a finite size surface, suggesting that this number fluctuates between different NPs. The mean number of motors $\langle N_m \rangle$ is related to the (mean) PEG-NLS-αβ-dynein anchoring distance ξ via $\langle N_m \rangle = 4\pi R^2/\xi^2$ or $\xi = \sqrt{4\pi}\, R/\sqrt{\langle N_m \rangle}$. Likewise, we may define a mean surface relative coverage $\theta = \left(\frac{\xi^*}{\xi}\right)^2$ describing the fraction of PEG-NLSs that end with a bound motor. In order to account for the fluctuations, we assume independent site (i.e. PEG-NLS) adsorption with probability θ for adsorption per site, suggesting that the random bound motor number obeys the well-known binomial distribution

$$P(N_m; \theta, N) = \frac{N!}{N_m!\,(N - N_m)!}\, \theta^{N_m} (1-\theta)^{N-N_m} \quad \text{(S16)}$$

where N is the number of PEG-NLSs, consistent with the mean number of motors obeying $\langle N_m \rangle = N\theta$.

However, since only NPs with $N_m \geq 1$ can bind to the MT, by assumption, we require the conditional binomial distribution $P^*(N_m; \theta, N) \equiv (P(N_m; \theta, N |\, N_m \geq 1)$, which is given by

$$P^*(N_m; \theta, N) = \frac{P(N_m; \theta, N)}{1 - (1-\theta)^N} \quad \text{(S17)}$$

Considering the mean number of motors among those NPs with $N_m \geq 1$ we find

$$\langle N_m^* \rangle = \frac{N\theta}{1-(1-\theta)^N} = \frac{\langle N_m \rangle}{1 - (1 - \langle N_m \rangle / N)^N} \quad \text{(S18)}$$

Given a value of θ, we can compute any mean of motility variable, $f(N_m)$, of the NP ensemble associated with this value of θ, e.g., velocity, run-time, and so on, as

$$\langle f(N_m) \rangle = \sum_{N_{m=1}}^{N} f(N_m) P^*(N_m; \theta, N) \quad \text{(S19)}$$

To examine the effect of the conditional binomial distribution on the observed motility, we first consider a characteristic value of $N = 4\pi R^2/{\xi^*}^2$ that corresponds to the estimated value of ξ^* of system I, $N \simeq 5$ (see Table 1 in the main text). Using this value of N, we depict in Figure S16 the theoretical ensemble average motility variables with θ ranging from 0.1 to 1, which determines $\langle N_m \rangle = N\theta$. We do that for results obtained for both MC-step-time and experimental time interval (0.27 s). We also show, for comparison, the theoretical (simulation results) for the case of a deterministic N_m. While deviations between the two calculations (obviously) do appear, they are not significant for most cases. The most pronounced differences appear in the MC-step-time longitudinal mean velocity for $N_m = 2{,}3$ (due to the contribution to the mean of the $N_m = 1$ population, whose velocity is high), which is strongly reduced for results obtained on 0.27 s time interval. To check the

sensitivity to the value of N, we also examine in Figure S17 the case of $N = 10$ (corresponding to the experimental systems II and III, see Table 1 in the main text). Similar to Figure S16, pronounced deviations appear in the MC-step-time longitudinal mean velocity for $N_{\mathrm{m}} = 2,3,$ and 4, but again the deviations are strongly reduced for results obtained for 0.27 s time interval. We conclude that while accurate comparison between experimental and theoretical results do require the (conditional) binomial distribution averaging, for approximate comparison with the current experimental results it is sufficient to consider deterministic values of N_{m}.

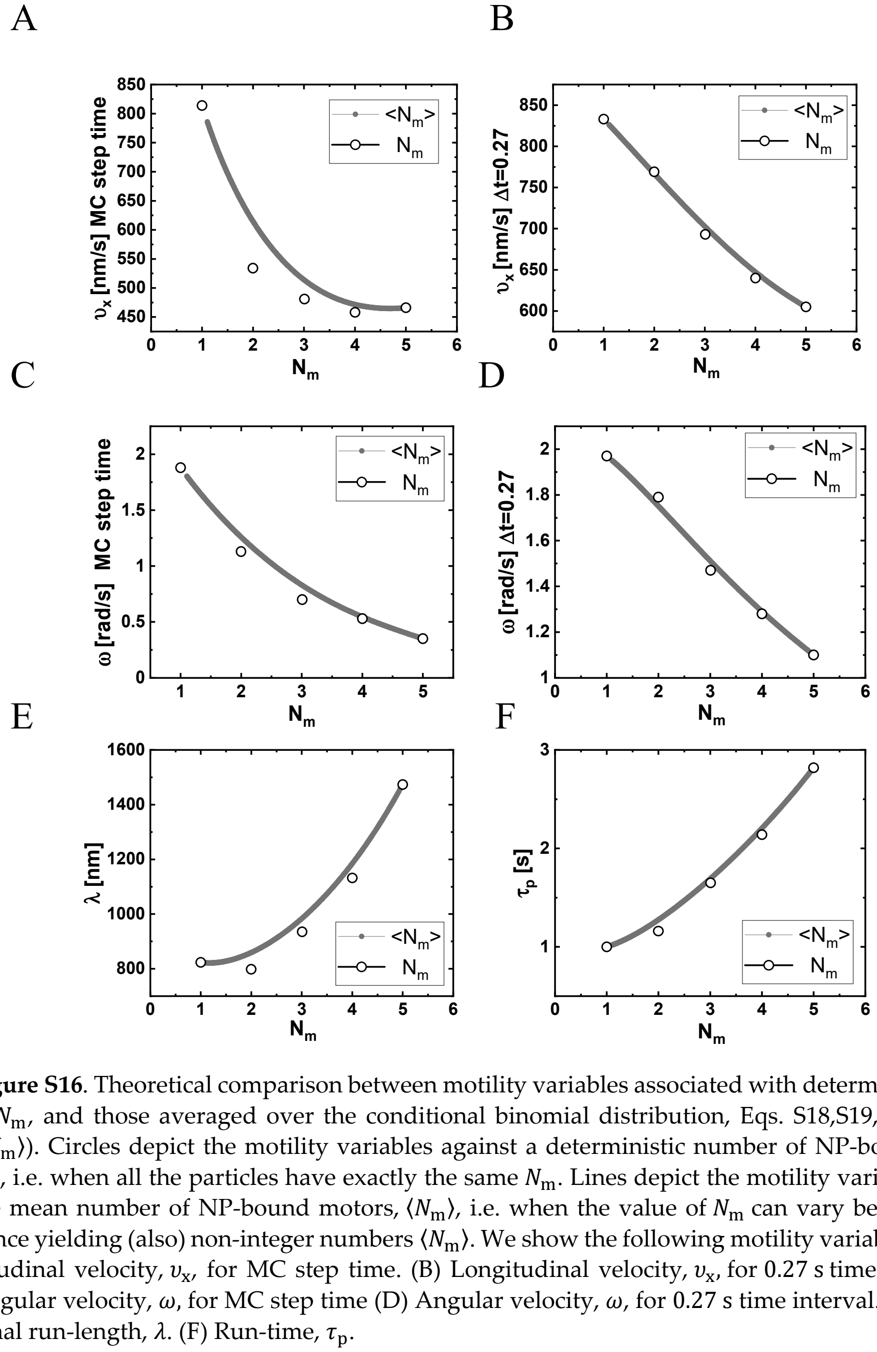

Figure S16. Theoretical comparison between motility variables associated with deterministic values of N_{m}, and those averaged over the conditional binomial distribution, Eqs. S18,S19, using $N = 5$ ($\langle N_{\mathrm{m}} \rangle$). Circles depict the motility variables against a deterministic number of NP-bound motors, N_{m}, i.e. when all the particles have exactly the same N_{m}. Lines depict the motility variables against the mean number of NP-bound motors, $\langle N_{\mathrm{m}} \rangle$, i.e. when the value of N_{m} can vary between NPs – hence yielding (also) non-integer numbers $\langle N_{\mathrm{m}} \rangle$. We show the following motility variables: (A) Longitudinal velocity, v_{x}, for MC step time. (B) Longitudinal velocity, v_{x}, for 0.27 s time interval. (C) Angular velocity, ω, for MC step time (D) Angular velocity, ω, for 0.27 s time interval. (E) Longitudinal run-length, λ. (F) Run-time, τ_{p}.

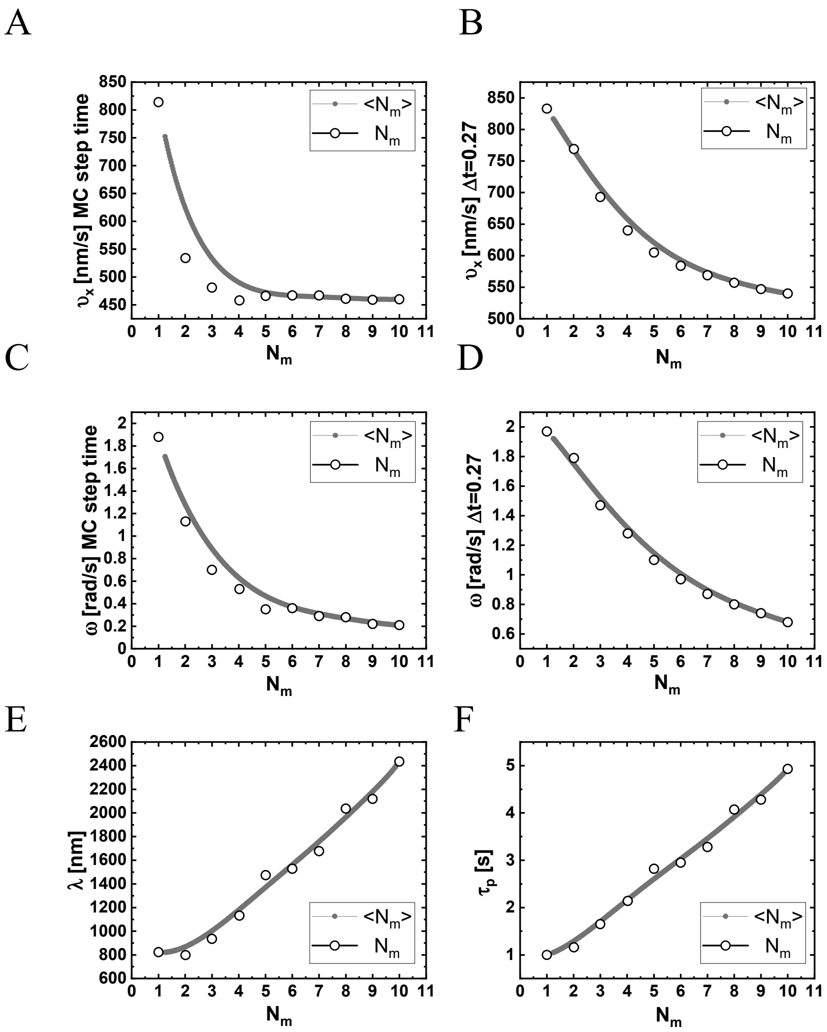

Figure S17. Same as Figure. S16 but for $N = 10$.

Movies

Movie S1 depicts the motion of NPs (green) on MTs (red) (system II). Some of the moving NPs are marked with white arrows (Bar is 5 μm).

Movie S2 depicts the motion of NPs (green) on MTs (red). Some of the moving NPs are marked with white arrows. In addition, one of the NPs move helically around the MT – this motion is marked by a pink arrow (system III). (Bar is 5 μm).

Movie S3 depicts the motion of a NP (green) which is marked by a white arrow and is hopping between crossing MT tracks (red) (system III). (Bar is 5 μm).

Movie S4 depicts the motion of a NP with a single NP-anchored motor ($N_{\mathrm{m}} = 1$).

Movie S5 depicts the motion of a NP with three NP-anchored motor ($N_{\mathrm{m}} = 3$).

Movie S6 depicts the motion of a NP with seven NP-anchored motor ($N_{\mathrm{m}} = 7$).

Movie S7 depicts the motion of a NP with 13 NP-anchored motor ($N_{\mathrm{m}} = 13$).
